# Deletion of neurturin impairs development of cholinergic nerves and heart rate control in postnatal mouse hearts

**DOI:** 10.14814/phy2.12779

**Published:** 2016-05-09

**Authors:** Anthony M. Downs, Hawa B. Jalloh, Kayla J. Prater, Santiago P. Fregoso, Cherie E. Bond, Thomas G. Hampton, Donald B. Hoover

**Affiliations:** ^1^ Department of Biomedical SciencesEast Tennessee State UniversityJohnson CityTennessee; ^2^ Mouse Specifics, Inc.FraminghamMassachusetts; ^3^ Center of Excellence in InflammationInfectious Disease and ImmunityQuillen College of MedicineEast Tennessee State UniversityJohnson CityTennessee

**Keywords:** Autonomic development, Baroreflex, cholinergic nerves, heart rate, neurotrophic factor

## Abstract

The neurotrophic factor neurturin is required for normal cholinergic innervation of adult mouse heart and bradycardic responses to vagal stimulation. Our goals were to determine effects of neurturin deletion on development of cardiac chronotropic and dromotropic functions, vagal baroreflex response, and cholinergic nerve density in nodal regions of postnatal mice. Experiments were performed on postnatal C57BL/6 wild‐type (WT) and neurturin knockout (KO) mice. Serial electrocardiograms were recorded noninvasively from conscious pups using an ECGenie apparatus. Mice were treated with atenolol to evaluate and block sympathetic effects on heart rate (HR) and phenylephrine (PE) to stimulate the baroreflex. Immunohistochemistry was used to label cholinergic nerves in paraffin sections. WT and KO mice showed similar age‐dependent increases in HR and decreases in PR interval between postnatal days (P) 2.5 and 21. Treatment with atenolol reduced HR significantly in WT and KO pups at P7.5. PE caused a reflex bradycardia that was significantly smaller in KO pups. Cholinergic nerve density was significantly less in nodal regions of P7.5 KO mice. We conclude that cholinergic nerves have minimal influence on developmental changes in HR and PR, QRS, and QTc intervals in mouse pups. However, cholinergic nerves mediate reflex bradycardia by 1 week postnatally. Deletion of neurturin impairs cholinergic innervation of the heart and the vagal efferent component of the baroreflex early during postnatal development.

## Introduction

The sympathetic and parasympathetic branches of the autonomic nervous system innervate the mammalian heart (Ardell 2001). These branches differ in their anatomy, neurotransmitters, and physiological role. Cardiac sympathetic neurons are located in sympathetic chain ganglia, which lie bilaterally alongside the vertebral column. Sympathetic neurons have long, axonal projections that innervate the heart and produce norepinephrine, which mediates cardioexcitatory effects. Cardiac parasympathetic neurons are located in intrinsic cardiac ganglia found on the surface of the heart. Axons from these neurons innervate the heart and produce acetylcholine (ACh), which mediates cardioinhibitory effects. The two divisions of the cardiac nervous system also differ in their developmental patterns, with the parasympathetic ganglia being smaller, more abundant, and forming a network on the surface of the heart. There is significant knowledge about the development, growth, and regional distribution of sympathetic nerve fibers in the heart (Ernsberger 2009; Ernsberger and Rohrer 2009), and nerve growth factor (NGF) has been identified as a crucial neurotrophic factor for the development of sympathetic neurons (Nam et al. 2013). However, much less is known about the development of cardiac parasympathetic neurons and nerves. Understanding the development of this system could lead to important insights into neuronal mechanisms contributing to heart dysfunction and cardiac disease.

Studies using transgenic mouse models have implicated neurturin (NRTN) as an important trophic factor for cholinergic neurons during development of the cardiac parasympathetic nervous system. NRTN is a member of the glial‐cell‐line‐derived neurotrophic factor (GDNF) family (Baloh et al. 2000; Kotzbauer et al. 1996). NRTN binds to a specific ligand binding protein complex consisting of GDNF family receptor *α*2 (GFR*α*2) and the transmembrane protein kinase, Ret. Deletion of *gfrα2* reduces cholinergic nerve fibers in the heart significantly compared to wild‐type (WT) mice (Hiltunen et al. 2000). Furthermore, deletion of *nrtn* reduces the number of cholinergic neurons in the intrinsic cardiac ganglia of adult mice by 65%, density of cholinergic nerve fibers in the sinoatrial (SA) node by 85%, and atrial ACh concentration by 74% (Mabe and Hoover 2009). Along with these neuroanatomical and neurochemical deficits, *nrtn* knockout (KO) mice have impaired chronotropic responses to vagal nerve stimulation but postjunctional responses to muscarinic agonists are unaffected (Mabe and Hoover 2009). Recent work has established that cholinergic ganglia form prior to birth in mice (Fregoso and Hoover 2012; Hildreth et al. 2008), but maturation of cholinergic cell bodies and most growth of cholinergic nerves in mouse heart occur during the first 3 weeks of postnatal life (Fregoso and Hoover 2012). Effects of NRTN deficiency on cardiac function and cholinergic innervation of the heart during this crucial period remain unknown.

In this study, we examined the effect of NRTN deficiency on developmental changes in heart rate (HR) and cardiac conduction (e.g., PR interval) in wild‐type (WT) and *nrtn* KO mouse pups ranging in age from postnatal day (P) 2.5 to 21. Electrocardiograms (ECGs) were obtained noninvasively from conscious, unrestrained mice. We demonstrated that a robust baroreflex‐mediated bradycardia had developed by P7.5 in WT mice but not in *nrtn* KO mice. Impaired baroreflex in the P7.5 *nrtn* KO mice was associated with marked deficiency of cholinergic nerves in the SA and atrioventricular (AV) nodes. Thus, our findings describe functional and neuroanatomical deficiencies in the heart of developing *nrtn* KO mice, further supporting an important role for NRTN in cardiac parasympathetic development.

## Materials and Methods

### Experimental animals

Male C57BL/6 mice (parents from Harlan, Indianapolis, IN) and *nrtn* KO mice (Kotzbauer et al. 1996) ranging in age from P2.5 to P21 were used for this study. Timed breeding of mice was performed, and the morning following birth was considered P0.5. All animal protocols were approved by the East Tennessee State University Animal Care and Use Committee and conformed to the guidelines of the National Institutes of Health as published in the Guide for the Care and Use of Laboratory Animals (Eighth Edition, National Academy of Sciences, 2011). The use of animals was minimized to the amount required to reach statistically valid conclusions.

### Genotyping

Deletion of *nrtn* was confirmed using the following protocol. Genomic DNA was extracted from mouse tail snips using the Qiagen Puregene Core Kit A (Qiagen, Valencia, CA) following the manufacturer's instructions. Briefly, 5 mm tail snips were incubated in cell lysis buffer with proteinase K (20 mg/mL) overnight at 50°C. After protein was precipitated, DNA was isolated from the supernatant by precipitation with 100% isopropanol, washed with 70% ethanol, air‐dried, and then resuspended in 50 *μ*L Tris buffer. Multiplex PCR was performed using Go Taq Hot Start Colorless Mastermix (Promega, Madison, WI), 250 ng genomic DNA, 5% DMSO, 0.8 *μ*mol/L forward primer, and 0.4 *μ*mol/L of each reverse primer in a total volume of 25 *μ*L. Primers used were as follows:

Forward: 5′‐CCGACGCGGTGGAGCTTCGAGAACTT‐3′;

Reverse WT: 5′‐AAGGACACCTCGTCCTCATAGGCCGT‐3′;

Reverse KO: 5′‐GAGATCAGCAGCCTCTGTTCCACATAC‐3′.

After 35 cycles of denaturation at 94°C for 30 sec, annealing at 63°C for 30 sec, and extension at 72°C for 1 min, PCR products were separated on a 2% TAE high‐resolution agarose gel with a 100 bp DNA molecular weight marker (Promega). Samples included a negative (no DNA) control, WT and KO. Positive samples yielded expected band sizes of 330 bp for WT and 200 bp for KO.

### Collection of ECG recordings

Electrocardiograms (ECGs) were recorded noninvasively from conscious, unrestrained neonatal and juvenile mice using the ECGenie apparatus (Mouse Specifics, Inc., Framingham, MA). This system comprises a recording box or platform with electrodes located in the floor, a preamplifier, and analog‐to‐digital conversion via Chart software version 5.2 (ADInstruments, Colorado Springs, CO). Prior to each recording session, mothers were removed from the home cage and placed in a new cage. A Gaymar T/Pump was used to keep the mice warm to prevent bradycardia during the absence of their mothers. Two different ECGenie chambers were used for recording the cardiac impulses of the mouse pups depending on their age. ECGenie was set to record cardiac electrical signals at 2 kHz. P2.5–P17 mice were placed in a small, heated recording box (The LifeSpoon), which had two electrodes for the mice that passively contacted their paws. The electrodes of LifeSpoon were moistened to improve electrical conductance. ECG recordings were obtained when the paws made contact with the electrodes. An elevated, 6.5 cm × 7 cm platform, with gel‐coated electrodes in the floor, was used to obtain ECG recordings from older mice. Serial ECGs were recorded between 8 and 9 am every day except weekends. After ECG recordings were collected, mice were weighed and transferred into a small plastic box that was lined with Kim‐Wipes and placed on a warming plate as stated above. Older mice were placed on an unheated recording platform for ECG recording.

### Analysis of ECGs

Several minutes of recording were collected from each mouse, and selected segments were analyzed using eMOUSE software (Mouse Specifics). Briefly, segments of interest from the recorded signals were highlighted and saved as text files. These text files were imported into e‐MOUSE and analyzed to determine HR and ECG intervals. This software uses peak detection algorithms to detect R waves, and a series of artificial intelligence algorithms to identify the PQRST morphology and interval durations.

### Tissue preparation and collection

Mice were deeply anesthetized with isoflurane and euthanized by decapitation. Hearts were removed quickly, washed in phosphate‐buffered saline (PBS), and immersion fixed in 10% neutral‐buffered formalin at 4°C for 24 h. Hearts were dehydrated and embedded in paraffin wax for histology. Paraffin‐embedded tissues were sectioned on the short axis, beginning anterior to the atria and continuing into the ventricular myocardium. Sections were cut at 10 *μ*m thickness using a Micron HM310 microtome and mounted on charged slides for immunohistochemistry.

### Immunohistochemistry

Sections were deparaffinized, rehydrated and immunostained at room temperature as described previously (Downs et al. 2014; Fregoso and Hoover 2012). Briefly, sections were washed in 0.1mol/L PBS, permeabilized with 0.4% Triton X‐100 in PBS containing 0.5% bovine serum albumin (BSA), and blocked 2 h in PBS containing 5% normal donkey serum (NDS), 1% BSA, and 0.4% Trition X‐100. Sections were then incubated for 16–18 h with a rabbit primary antibody to the vesicular acetylcholine transporter (VAChT; Synaptic Systems, Gottingen, Germany), a specific cholinergic marker, diluted 1:1000 in blocking buffer. Sections were washed again in PBS, permeabilized, blocked in 5% NDS, and then incubated for 2 h with donkey anti‐rabbit IgG conjugated to AlexaFluor 555 (1:200; Invitrogen, Carlsbad, CA). Sections were washed several times in PBS, and coverslips were attached using Citifluor mounting medium (Ted Pella Inc., Redding, CA). Slides were then sealed with clear nail polish.

### Microscopy and image analysis

Immunostained slides were viewed using an Olympus BX41 microscope. Digital images were obtained using an Olympus Qcolor 3 camera under fluorescence illumination. Nerve fiber densities in the SA node and AV node were evaluated using ImageJ software (NIH, Bethesda, MD). Multiple representative sections for each area of interest were evaluated. Nerve fiber densities were reported as percentage of total area evaluated (% area).

### Evaluation of baroreflex

Baroreflex function was evaluated in P7.5 WT and *nrtn* KO mice using atenolol and phenylephrine at doses proven effective in other studies (Just et al. 2000; Quigley et al. 2005, 1996; Sato 2008). Drugs were dissolved in sterile filtered saline and administered by intraperitoneal (i.p.) injection. Drugs were given in a volume of 50 *μ*L using 0.3 cc insulin syringes with siliconized 28 g needles. Syringes were placed on a heating pad well before injection to assure that the fluids were warm. After collecting baseline ECGs, 5 mg/kg atenolol was given. The response to atenolol was determined 20 min later by recording the ECG for 3–4 min. Phenylephrine (3 mg/kg) was given 25 min after atenolol, and the ECG was recorded immediately for 5 min.

### Statistical analysis

Data are presented as the mean ± SEM. Since WT and KO mice were born on different days, time points were not matched completely. Only matched time points were used when making between strain comparisons. All statistical analyses were performed using GraphPad Prism 6 (GraphPad Software, Inc, La Jolla, CA). Data collected from the same WT and KO mice over time were compared at matched time points using two‐way ANOVA for repeated measured. Sidak's multiple comparison test was used to identify between group differences at matched times. Unpaired *t*‐tests were used to compare WT versus *nrtn* KO nerve density data and to make between groups comparisons of data in the baroreflex experiment. Within groups, comparisons were made using one‐way ANOVA for repeated measures. *P *<* *0.05 was considered significant.

### Drugs

Atenolol (A7655), and (‐)‐phenylephrine (P6126) were obtained from Sigma‐Aldrich (St. Louis, MO).

## Results

### Neurturin KO mice

NRTN‐deficient pups appeared similar to WT pups in both appearance and behavior. Weight was measured daily during the study period, and both strains gained weight equally.

### Postnatal changes of cardiac rate and conduction

**Figure 1:**
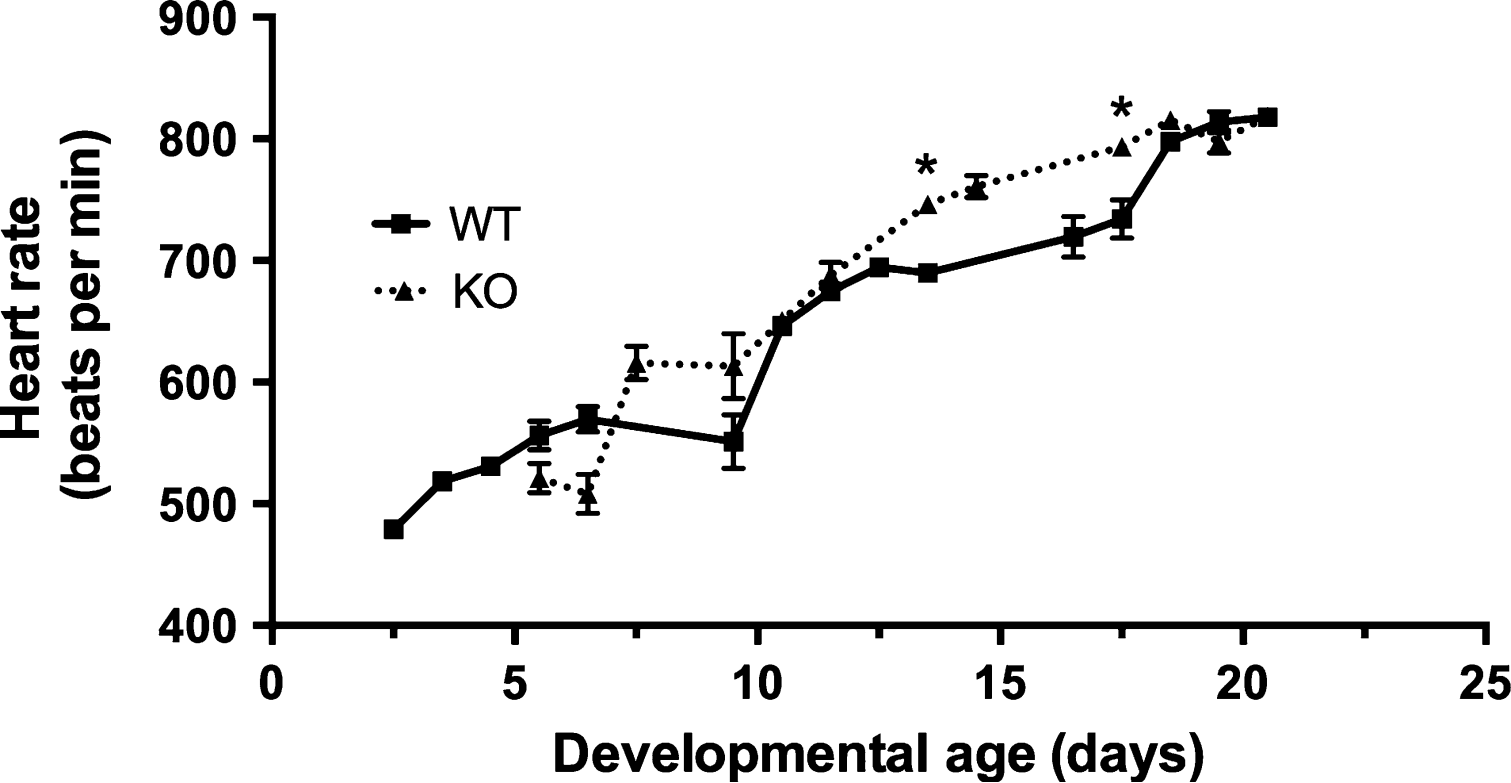
Heart rate increases postnatally in WT and *nrtn* KO mice. Heart rate increased from P2.5 and reached a peak around P18.5. Data collected from the same WT and KO mice over time were compared at matched time points using two‐way ANOVA for repeated measures. This analysis showed significant effects of time (*F*_6,72 _= 95.0, *P *<* *0.0001), mouse strain (*F*_1,12 _= 9.11, *P *<* *0.05) and a time–strain interaction (*F*_6,72 _= 2.80 *P *<* *0.05). Post hoc testing with Sidak's multiple comparison test showed that heart rate was significantly higher (*) in *nrtn* KO mice than WT at P13.5 (WT 689 ± 7 bpm, KO 746 ± 7 bpm) and P17.5 (WT 734 ± 16 bpm, KO 794 ± 4 bpm). Values graphed are means ± SEM,* n* = 5–14 per group. KO, knockout; WT, wild‐type.

HR increased between P2.5 and P21 for both WT and *nrtn* KO mice (Fig. 1). The beating rate was significantly higher in *nrtn* KO mice than WT at P13.5 (746 ± 7 bpm for KO versus 689 ± 7 bpm for WT) and P17.5 (794 ± 4 bpm for KO versus 734 ± 16 bpm for WT). No other significant differences between WT and KO HRs were detected for matched time points in this experiment. However, HR was also higher in KO pups at P7.5 in later experiments aimed at evaluating the baroreflex. HR reached a peak around P18.5 (815 ± 5 bpm for KO versus 798 ± 7 bpm for WT).

**Figure 2:**
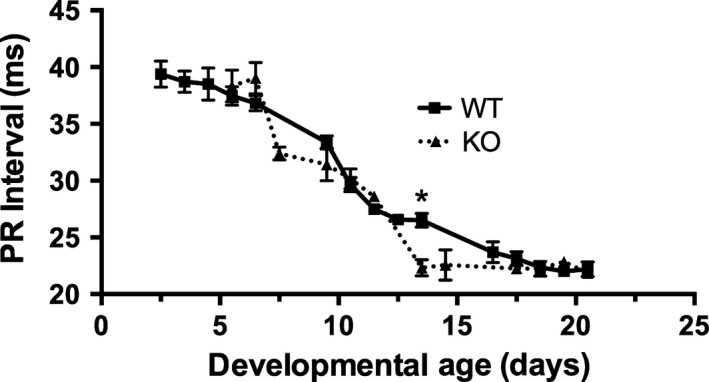
PR interval decreases with developmental age in both WT and *nrtn* KO mice. PR interval decreased from P2.5 to about P17.5. Data collected from the same WT and KO mice over time were compared at matched time points using two‐way ANOVA for repeated measures. This analysis showed significant effects of time (*F*_6,72 _= 80.3, *P *<* *0.0001) and a time–strain interaction (*F*_6,72 _= 3.94, *P *<* *0.005). Mouse strain alone did not have a significant effect (*F*_1,12 _= 4.24, *P *>* *0.05). Post hoc testing with Sidak's multiple comparison test showed that PR interval was significantly shorter (*) in *nrtn* KO than WT mice at P13.5 (WT 26.5 ± 0.6 msec, KO 22.5±1.3 msec). Values graphed are means ± SEM,* n* = 5–15 per group. KO, knockout; WT, wild‐type.

PR interval decreased in WT and KO mice during the period from P2.5 to about P14.5–17.5 (Fig. 2). PR interval was significantly less in KO animals than WT animals at P13.5. (26.5 ± 0.6 msec for WT versus 22.3 ± 1.3 msec for KO mice). However, there was not a significant difference in PR interval between WT and KO animals at other matched developmental ages. QRS and QTc intervals showed similar, age‐dependent decreases in both groups and reached adult values at or before P21 (data not shown).

### Deletion of nrtn reduces cholinergic innervation of the heart

**Figure 3:**
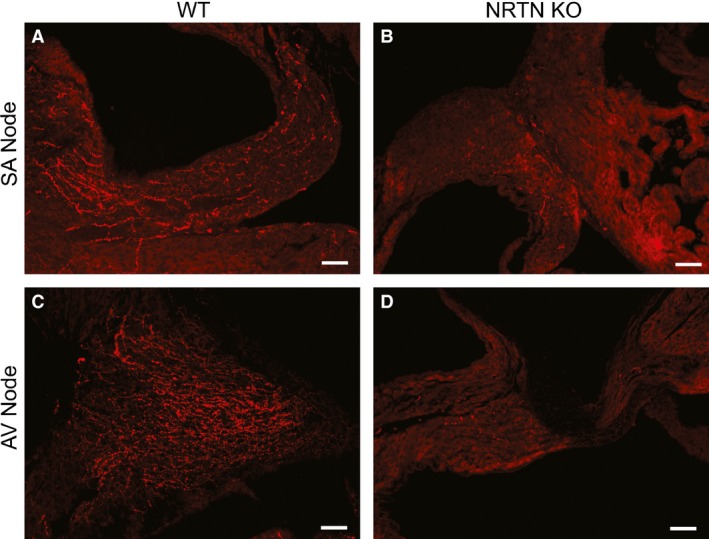
Photomicrographs of the SA node and AV node in WT and *nrtn* KO mice at P7.5 that were stained for vesicular acetylcholine transporter (VAChT). VAChT‐positive fibers were abundant in the SA node of WT mice (A) but sparse in *nrtn* KO hearts (B). The same pattern was observed in the AV node (C & D). Scale bar is 50 *μ*m. AV, atrioventricular; KO, knockout; SA, sinoatrial; WT, wild‐type.

**Figure 4:**
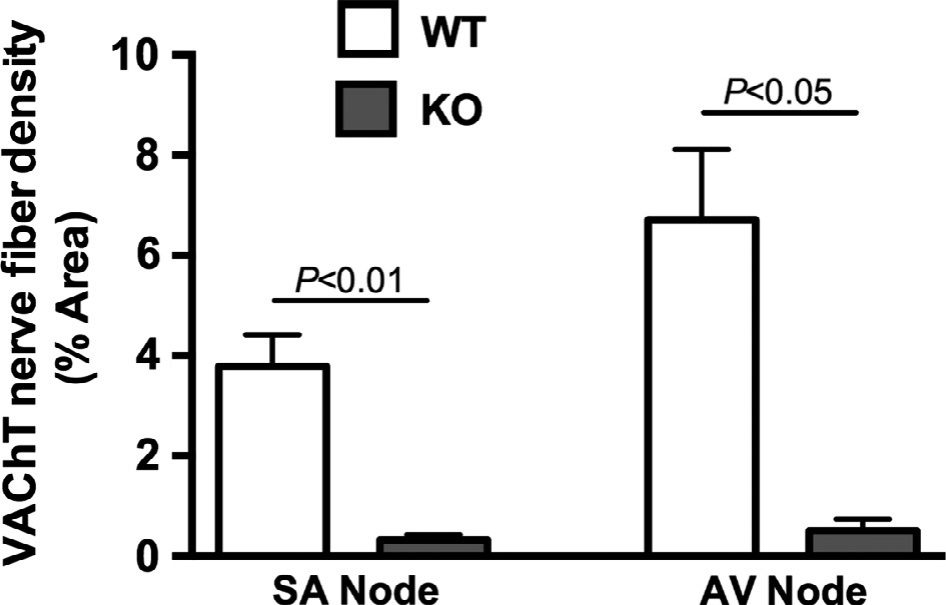
*Nrtn* KO mice have reduced cholinergic innervation of the SA and AV nodes at P7.5. Quantification of nerve fiber density at the SA node and AV node of P7.5 WT and *nrtn* KO mice was performed using ImageJ to analyzed digital images of immunostained sections. VAChT‐positive nerve fibers are significantly less abundant in the nodes of *nrtn* KO animals. The AV node has more cholinergic innervation than the SA node at P7.5. Values are means ± SEM, *n* = 3 per group. AV, atrioventricular. KO, knockout; SA, sinoatrial; WT, wild‐type.

We evaluated cholinergic innervation of the SA and AV nodes of P7.5 mouse pups using immunohistochemistry for the cholinergic marker VAChT. In agreement with our previous findings (Fregoso and Hoover 2012), we observed abundant VAChT‐immunoreactive nerve fibers in the SA node (Fig. 3A) and AV node (Fig. 3C) of P7.5 WT mice. In marked contrast, cholinergic nerve fibers were sparse in the same regions of *nrtn* KO mice at this developmental age (Fig. 3B and D). Quantitative evaluation of cholinergic nerve fiber density established that values are significantly lower in nodal tissue of KO pups compared to WT (Fig. 4; SA node: 3.8 ± 0.6% area for WT versus 0.32 ± 0.11% area for KO, *n* = 3, *P *<* *0.01; AV node: WT 6.7 ± 1.4% area for WT versus 0.50 ± 0.24% area for KO, *n* = 3, *P *<* *0.5).

### Nrtn deletion causes impaired baroreflex function

**Figure 5:**
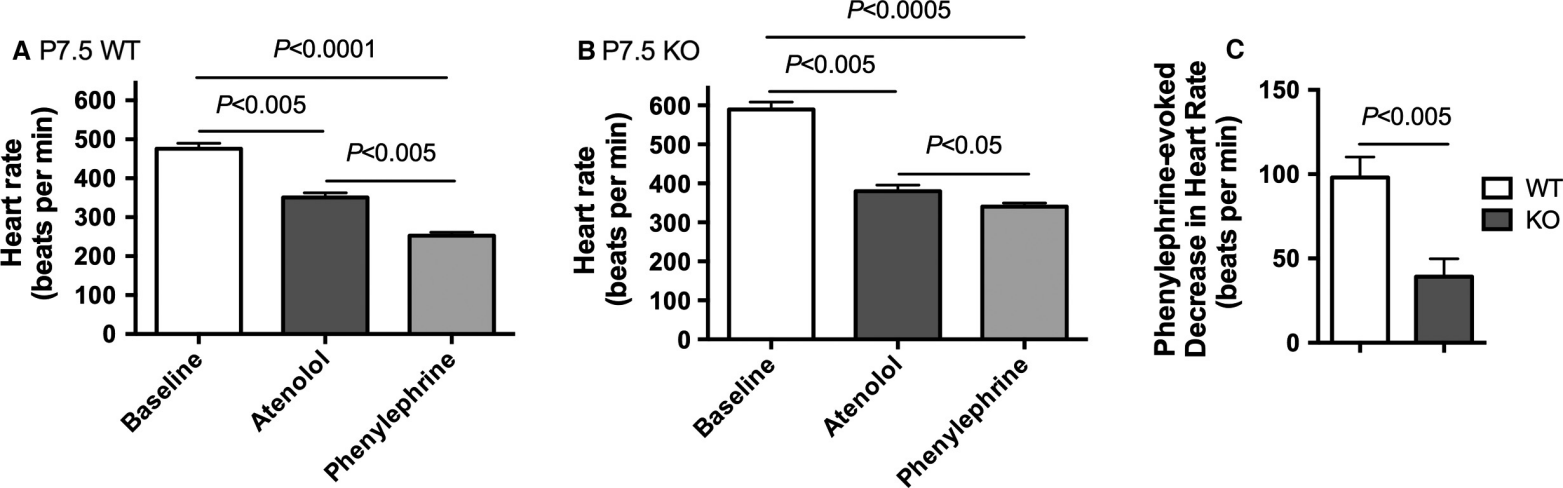
The cholinergic component of the baroreflex is impaired in *nrtn* KO mice at P7.5. The baroreflex was evaluated by injecting mice with atenolol followed by phenylephrine 25 min later. Baseline HR was higher in KO mice compared to WT mice. Atenolol reduced HR in both groups, and HRs did not differ between groups after this treatment. Phenylephrine decreased HR in both groups, but the absolute and percent reductions were significantly larger in the WT group. Values are means ± SEM, *n* = 6 per group. (A and B) Data for each group were evaluated by repeated measures ANOVA, followed by Tukey's multiple comparisons test. (C) Decreases in HR after treatment with phenylephrine were compared using an unpaired *t*‐test.KO, knockout; HR, heart rate; WT, wild‐type.

We stimulated the baroreflex in WT and *nrtn* KO animals at P7.5 to assess cardiac parasympathetic nervous system function. Baseline HR was significantly higher in KO mice at this age (Fig. 5A and B; 475 ± 14 bpm for WT versus 589 ± 19 bpm for KO, *n* = 6 per group, *P *=* *0.0007). Mouse pups where first treated with the *β*_1_ adrenoceptor antagonist atenolol to prevent potential decreases in HR due to sympathetic withdrawal. Atenolol, given at a dose of 5 mg/kg (i.p.), reduced HR significantly for over 30 min (data not shown). To evaluate the baroreflex, we injected both WT and *nrtn* KO mice with atenolol followed by an injection of phenylephrine (3.0 mg/kg, i.p.) 25 min later. Atenolol decreased HR significantly in both WT and *nrtn* KO animals (Fig. 5A and B). HR values after atenolol treatment did not differ between groups (351 ± 12 bpm for WT versus 380 ± 16 bpm for KO, *n* = 6 per group, unpaired *t*‐test, *P *=* *0.1677). Treatment with phenylephrine reduced HR further in WT mice (Fig. 5A) but to a much smaller degree in KO mice (Fig. 5B, and C). This finding, in combination with the observed deficit of cholinergic nerve fibers, shows that vagal efferent baroreflex function is impaired in *nrtn* KO animals.

## Discussion

Results of this study show that baseline HR and PR interval undergo respective, time‐dependent increases and decreases in mouse pups between the ages of P2.5 and P21. Deficiency of the neurotrophic factor NRTN, which is required for development of normal cholinergic innervation of the adult heart, has minimal effect on developmental changes in these cardiac parameters or on QRS and QTc intervals. Treatment with atenolol, a selective *β*_1_‐adrenergic receptor antagonist, reduced baseline HR significantly in WT and KO pups as early as P7.5, suggesting that adrenergic mechanisms already have a dominant role in regulation of cardiac function at this early age. However, further pharmacological testing revealed that a prominent vagal baroreflex could be elicited at P7.5 in WT pups. Immunohistochemical experiments confirmed that abundant cholinergic nerves, which mediate vagal bradycardia, had developed by this time point in the SA and AV nodes of WT mice. A deficit of cholinergic nerves and impaired reflex vagal bradycardia occurred in *nrtn* KO pups, confirming that bradycardia in the WT pups was mediated by endogenous acetylcholine.

Our findings concur with previous studies, which showed postnatal increases in HR in mice (Heier et al. 2010; Sato 2008) and decreases in heart period in rats (Quigley et al. 1996), although some of these investigators used recording methods that are more cumbersome or intrusive. The age‐dependent increase in HR is predominantly due to tonic sympathetic nerve activity since our results and previous work in mouse and rat pups demonstrated that *β*‐adrenergic receptor blockade causes significant decreases in baseline HR. Furthermore, previous studies have established that autonomic innervation of the heart develops postnatally in mice and rats (Fregoso and Hoover 2012; Nyquist‐Battie et al. 1994). Both cholinergic and adrenergic nerves grow most rapidly during the first two postnatal weeks and approximate the density found in adult hearts by P21. In fact, we found that baseline HR in P21 mice was comparable to that reported for adult mice using the same recording technology (Hoover et al. 2015; Mabe and Hoover 2011). Certainly, the handling of pups and adult mice, which is required for placement of the mice on the noninvasive ECGenie recording platform, can cause activation of the sympathetic nervous system, reflected by baseline HRs that are higher than observed in studies that use telemetric methodology (Ishii et al. 1996; Just et al. 2000). While sympathetic mechanisms undoubtedly play a role in the developmental increase in HR in neonatal mice, we cannot exclude some contribution from developmental changes in the SA node and the intrinsic HR. However, this seems unlikely since HRs did not differ between WT and KO pups at P7.5 after treatment with atenolol.

Deletion of *nrtn* does not affect development of sympathetic neurons (Heuckeroth et al. 1999; Hiltunen et al. 2000; Mabe and Hoover 2009) and had limited effects on the development of HR and PR interval in neonatal mice. This finding undoubtedly reflects a dominant role of sympathetic mechanisms in regulation of cardiac function in neonatal mice, as previously established for adults (Ishii et al. 1996; Just et al. 2000). Furthermore, treatment of mice with atropine does not cause an increase in heart rate (Just et al. 2000), which does occur in larger mammals. Nevertheless, we did observe time points during development when KO mice had slightly higher HRs (P7.5, P13.5 and P17.5) and a shorter PR interval (P13.5). These differences may be due to the deficiency of cholinergic nerves and a resulting enhanced sympathetic response at these times. The isolated occurrence of these differences could be caused by the dynamic nature of the system during development due to differences in the rate of sympathetic and parasympathetic nerve growth and immaturity of neural circuitry. However, we cannot exclude the possibility that these differences are unrelated to the cholinergic deficit in KO mice.

While sympathetic mechanisms have a dominant role in regulating baseline cardiac function in neonatal mice, our data show that cholinergic mechanisms for regulation of HR are well established by the first week of postnatal life. This observation concurs with previous studies done with conscious and anesthetized rat pups (Ishii et al. 2001; Quigley et al. 2005). Additionally, we established that the SA node had developed a dense cholinergic innervation at this postnatal age. Previous studies showed that preganglionic vagal input to the cardiac parasympathetic ganglia develops before the cholinergic neurons send axonal projection to the heart (Fregoso and Hoover 2012; Hildreth et al. 2008). Collectively, this work demonstrates that the vagal efferent circuit and corresponding baroreflex mechanisms are established and functional early during postnatal development of mice. In contrast, cholinergic nerve fibers were sparse in the SA node of P7.5 *nrtn* KO mice, and reflex bradycardia was substantially less in these animals. This finding is consistent with previous work showing that adult nrtn KO mice have impaired chronotropic responses to electrical stimulation of the vagus nerve (Mabe and Hoover 2009). Although we cannot exclude the possibility that deletion of NRTN affects the afferent limb of the baroreflex, this scenario seems unlikely since nrtn (−/−) mice have the normal numbers of neurons in the nodose ganglia (Baloh et al. 2000).

Our observation of dense cholinergic innervation of the AV node at P7.5 suggests that cholinergic regulation of AV condition is likewise established early in the mouse heart. The sparsity of cholinergic nerves in the AV node of KO mice at the same developmental age suggests that cholinergic regulation of AV conduction would be impaired.

In conclusion, our findings confirm that substantial changes in cardiac rate and conduction occur in mice during the first 3 weeks of postnatal life, which is the time interval for the development of cardiac adrenergic and cholinergic nerves. Sympathetic mechanisms have a dominant role in regulation of HR in the neonatal mouse, as in adults. NRTN is required for the development of cholinergic nerves in the mouse heart, but the deficiency of cholinergic nerves in nodal tissue has very little impact on the changes in basal cardiac rate and conduction that occur during the first 3 weeks of postnatal life. However, the deficit of cholinergic nerve fibers in the hearts of *nrtn* KO mice does affect dynamic control of HR by the parasympathetic nervous system, as evidenced by the impaired negative chronotropic response to baroreceptor activation evoked by phenylephrine in P7.5 pups. NRTN deficiency does not appear to affect development of cardiac dromotropic properties since postnatal changes in PR, QRS and QTc intervals were very similar in WT and KO mice.

## Conflict of Interest

TGH is owner of the company that has commercialized the ECGenie recording instrumentation described in Methods.
